# Dimensions of Attitudes towards the Mentally Ill in the General Population Stability and Change over Time at Urban and Rural Sites

**DOI:** 10.1155/2013/319429

**Published:** 2013-03-07

**Authors:** Tom Sørensen, Andreas Sørensen

**Affiliations:** ^1^Division of Mental Health and Addiction, Institute of Clinical Medicine, University of Oslo, 0316 Oslo, Norway; ^2^Division of Research, North Coast Psychiatry, 1482 Nittedal, Norway

## Abstract

Items measuring attitudes toward the mentally ill can be limited in relevance to a particular period or place. The main objective of the study was to provide evidence toward a questionnaire that was short and psychometrically stable over time and geography, and that could be used within comprehensive mental health surveys of general populations. Four rural samples, Lofoten 1983 (*n* = 470), 1990 (*n* = 947), 2000 (*n* = 864), and Valdres 2010 (*n* = 772), and two urban samples, Oslo 1990 (*n* = 948) and 2000 (*n* = 467), were used to test this. The questionnaire was self-administered with fixed questions and response alternatives. Using the three Lofoten and the two Oslo samples, the stability of the factor analytic structure of 19 attitude items was established. In all analyses, there was a clear leveling off after three factors. The 13 highest loading items on these three factors were used in a new rural region, Valdres, in 2010. The three established factors/dimensions, named Distance, Demands, and Positive, seemed to be reasonably stable within a variety of Norwegian samples. On the other hand, the analyses were different enough to recommend researchers and politicians to be careful when comparing absolute levels of the suggested indexes across different locations and at different points in time.

## 1. Introduction

Attitudes towards people who are mentally ill are part of the context that influences the quality of life of those already sick, people with mental problems in the general population that are portraying help-seeking behavior, but not yet in treatment, and the willingness to use resources on psychiatric services. Historically, the interest in and measurement of attitudes toward the mentally ill was linked to the rise of social psychiatry as a frame of reference [1]. The first major studies in the early 1950s were done in an atmosphere of a medical psychiatric model, and the aim was to have people look upon mental illness as any other disease (somatic diseases having a higher prestige). Publications in the 1970s and 80s also focused on labeling theories [2, 3]. A seminal study, both regarding methods and practical development of community psychiatry, was the neighborhood focus of Taylor and Dear [4]. The present studies could likewise be seen as a component in the development of a community approach to psychiatry, for example, treating patients closer to their home milieu.

Before establishing a community mental health centre in the Lofoten Islands (one of the rural sites in this paper) in 1983, a mental health survey was carried out on a representative sample of the local general population. This study has been used as feedback for clinical and preventive work and as basis for following the Lofoten population over time in a community psychiatric context [5–12]. Further surveys were done in 1990 and 2000. In addition to Lofoten, the surveys also included an urban site, a borough in Oslo, the capital of Norway. The 1990 and 2000 studies in Oslo and Lofoten were joined together by the ability to identify respondents that participated at both points in time, that is, making possible studies of change linked at the individual level [13–17].

There were two reasons for the inclusion of measurements of people's attitudes toward the mentally ill in the Lofoten 1983 population, that is, (1) to detect characteristics of groups with high and low tolerance for the mentally ill as basis for community education and (2) to have a basis for monitoring possible changes in attitudes during the development of the new community approach to mental health services. These population-directed motives had much in common with the public attitudinal approach that Madianos et al. [18] had in their Athens study from about the same period. In Lofoten, especially during the late 80s and in the 90s [5, 19], a multitude of undertakings at the community level, related to enhance more tolerant attitudes towards people with mental health problems, were implemented. On the other hand, the main influential factors over time for the study in Oslo and the Valdres region were the general change in psychiatric services and highlighting psychiatry and psychiatric patients in the media.

## 2. Aims

The main objective of the study was to develop instruments about attitudes toward the mentally ill that are short and psychometrically stable over time and geography and that could be used in comprehensive mental health surveys of general populations. The aim was to find items/indexes that could validly be compared over time and between sites; that is, the same items should load high on the same factors across studies. Theoretically, some items may be limited in their relevance to a particular period and/or place of study. In this paper, we used four rural samples, Lofoten 1983, Lofoten 1990, Lofoten 2000, and Valdres 2010, and two urban samples, Oslo 1990, and Oslo 2000, to test this.

1. To analyze the stability of the factor analytic structure of 19 attitude items over time at a rural and an urban site, the three Lofoten, and the two Oslo samples were utilized.
2. When/if such stability across place and time is established, select items to be used in future surveys.
3. Compare the factor structure using only the chosen items in the three Lofoten, the two Oslo, and a new sample from a different Norwegian region (10 years after the last Lofoten-Oslo surveys).
4. If the items revealed in the initial Lofoten-Oslo factor analyses are used as indexes in the six samples, what are the scale reliabilities?

## 3. Material and Methods

### 3.1. Study Sites

The first five surveys cover one rural and one urban site [9, 15, 20]. Lofoten, the rural site, is an area dominated by fishing, fishing industry, and agriculture. Before establishing the psychiatric outpatient clinic at the rural site, Lofoten, people usually had to travel 6–12 hours by car and boat for psychiatric consultation or inpatient treatment, but already before the first study period (1983), this could be shortened by the use of airplane. Historically, before 1983, for most people in Lofoten, psychiatry meant being hospitalized for a severe psychiatric condition, often for a long period of time, far away from the region. Hence, the introduction of a community mental center was welcomed as a resource for treatment and consultation on a higher professional level, also for psychiatric problems that had previously received little attention or had been treated at a lower professional level. The urban site, Søndre-Nordstrand in Oslo (borough in the capital of Norway), was chosen because it represented inhabitants in a large city. Oslo had already for many years, even before 1990, had outpatient services for psychiatric patients. The district of Oslo where the study took place had already had a regional mental health centre for some years before the first survey. Between 1990 and 2000, the regional center started to provide low-threshold in-patient services for psychiatric patients. The sixth site, Valdres [21], is a rural mountainous region in South Norway. In 2010, it had a local outpatient clinic and active psychiatric service, also at the municipal level.

### 3.2. Samples (See Table 1)

The first five samples were representative population samples (populations 18 years or older). The respondents were interviewed by a structured questionnaire in their own homes. The first part of the interview in the first five samples was a self-administered questionnaire with fixed questions and response alternatives. The interviewers administered the questionnaire but did not verbally ask these questions. The attitude items were within this first section. The order of topics in this section was the same for all five samples. In the sixth sample, Valdres 2010, the respondents also were required to fill in a self-administered questionnaire with fixed questions and response alternatives.

In the Lofoten 1983 sample [20], 470 people, 18 years or older in the four Lofoten municipalities that constituted the catchment area for the Lofoten community psychiatric center (Vågan, Vestvågøy, Flakstad, and Moskenes), were interviewed. They were randomly picked from the census of the four municipalities by the Norwegian Bureau of Statistics. This represents 80% of those who were eligible for inquiry from the original drawn sample.

The 1990 and 2000 Lofoten and Oslo samples were also drawn randomly by the Norwegian Bureau of Statistics from the actual populations, 18 years and older. Hence, in 1990, a random sample of 2727 individuals was drawn [22]. Of these, 713 refused to participate, leaving 2014 participants, 1009 in Oslo and 1005 in Lofoten. Thus, the response rate was 74%. The respondents from 1990 were also approached in 2000. In 2000, an additional new random sample (500 people from Lofoten and 500 from Oslo) was drawn. The response rate of the combined Oslo-Lofoten 2000 was 52% of the eligible sample. Respondents that had moved from the study regions in Lofoten and Oslo were excluded from the analyses, leaving a sample of 483 in Oslo and 890 in Lofoten.

In Valdres 2010 [21], a two-step sampling procedure was carried out. In the first step, each of the six municipalities in the Valdres region (Sør-Aurdal, Nord-Aurdal, Vestre Slidre, Øystre Slidre, Vang, and Etnedal) chose two local communities; that is, in all, 12 local communities were selected. The survey was to constitute a basis for later mental health promotion efforts. In the 12 local communities, 2325 questionnaires were distributed to people 18 years or older. The questionnaires were delivered to all households where people were physically present in the period and collected about one week later; hence, the available universe was closer to a total, rather than a randomly drawn sample. In all, 925 questionnaires were collected, that is, a response rate of 40%. 

The sizes of the six samples with complete data sets for factor analyses of the attitude items were as follows.

### 3.3. Questionnaire Attitudes towards the Mentally Ill

A mental health survey was carried out before the start of a community mental health center in Lofoten in 1983. The questionnaire included items pertaining to the general population's attitudes toward the mentally ill [23, 24]. The point of departure for this part of the questionnaire was a study by Taylor and Dear [4]. The study was seen as particularly relevant because of its practical aim towards establishing mental health facilities in residential areas. Sixteen of the 37 items in the Lofoten 1983 attitude scale were taken from Taylor and Dear's questionnaire (CAMI), selecting those items that had the highest loadings on the four factors obtained in the analyses of the Canadian data. These items were intended to cover authoritarianism, benevolence, social restrictiveness, and community mental health ideology. The additional 21 items were designed particularly for the Lofoten 1983 study. All 37 items employed a five-point agree-disagree scale. Some of the additional items could be grouped under the same four headings as in the Taylor and Dear study. In addition, items were included that covered demands for behavior and skills that patients should meet before they were allowed to settle down outside a psychiatric hospital, as well as popular stereotypes of patients being dangerous and not to be trusted. Demands for skills and behavior if psychiatric patients should live in ordinary neighborhoods were important issues in the professional debate of this period.

Taylor and Dear claimed to have four factors. However, looking closely at the factor analyses in the Canadian study, it rather indicated a three-factor solution. The analyses of the 16 Taylor and Dear items in the Lofoten 1983 sample also yielded at most three factors, with even some evidence in favor of only two meaningful factors. The analyses of the Lofoten 1983 sample took into account the problems with acquiescence in the questionnaire [23]. Hence, when the 37 attitudes items in the questionnaire were analyzed, it showed two factors resembling the results from Taylor and Dear and an additional third factor pertaining to demands toward mentally ill people, that is, some of the new added items particular for Lofoten 1983.

The questionnaires in 1990 and 2000 (Lofoten and Oslo) had 19 items (see Appendix A), and all of them were among the 37 used in the 1983 survey. The items for the later Lofoten and Oslo surveys were selected by factor analyses (i.e., principal components procedures) of the Lofoten 1983 sample. The items loading highest on three rotated (oblique oblimin) factors and on the first unrotated factor were chosen. Ten of the items came from the original Taylor and Dear scale (see Appendix A; marked (T)). The selection of 13 items in the Valdres 2010 survey (see Appendix A; marked (V)) was based on factor analyses of the five earlier studies in Lofoten and Oslo (see Results section).

## 4. Analyses

The first sets of analyses explored the factor structures of the 19 common attitude items of the first five samples (Lofoten and Oslo). Analyses of factorial invariance often employ confirmatory factor analysis, testing whether an a priori defined structure can be replicated in different populations (e.g., see Cheung and Rensvold [25]). At this point, it was a choice between multi sample analyses and a series of separate analyses of each sample. The study did not have definite models, and the confirmatory approach was believed to be somewhat premature with regard to measures of attitudes toward psychiatric patients. Hence, the study relied mainly on exploratory factor analyses, but partly in combination with confirmatory analyses (see later). Five sets of exploratory factor analyses were carried out, that is, Lofoten 1983, Lofoten 1990, Lofoten 2000, Oslo 1990, and Oslo 2000. The extraction method was maximum likelihood. Decision about number of factors relied mainly on comparing scree plots. Oblimin rotation was employed. The oblique solution was chosen because, theoretically, all the attitudinal dimensions were expected to reflect some general positive or negative attitude toward people who are mentally ill. The stability of the factor structure was assessed by using a combination of exploratory and confirmatory factors analyses. First, for each of the five samples (e.g., Lofoten 1983), an exploratory factor analysis on the pooled data from the four other samples was carried out. Then, it was tested whether the factor structure in each sample deviated significantly from that defined by the analysis of the four other samples, using confirmatory factor analysis procedures. In addition to the chi-square test, two of the most commonly used descriptive fit measures are presented, that is, the comparative fit index (CFI) and the root mean square error of approximation (RMSEA) [26]. Since a five-category response scale was used, the data were most appropriately regarded as ordinal. The variable values were therefore transformed to normal scores, and factor analyses were carried out on these scores. The PRELIS data program was used [27]. The exploratory factor analyses were carried out using the SPSS program and confirmatory analyses with the LISREL program.

The 13 highest loading/stable items of the three factors revealed in the factor analyses of the five surveys from Lofoten and Oslo were used in the Valdres 2010 survey. Still being in an exploratory phase, all six samples were separately factor analyzed (using the 13 chosen items). These analyses were done in STATA, version 12. The extraction method was principal factors, and a retained three-factor oblimin rotation was performed. 

## 5. Results

### 5.1. Aims 1 and 2: Factor Structure of the 19 Common Attitude Items Used in Lofoten 1983, Lofoten 1990, Oslo 1990, Lofoten 2000, and Oslo 2000

**Figure 1:**
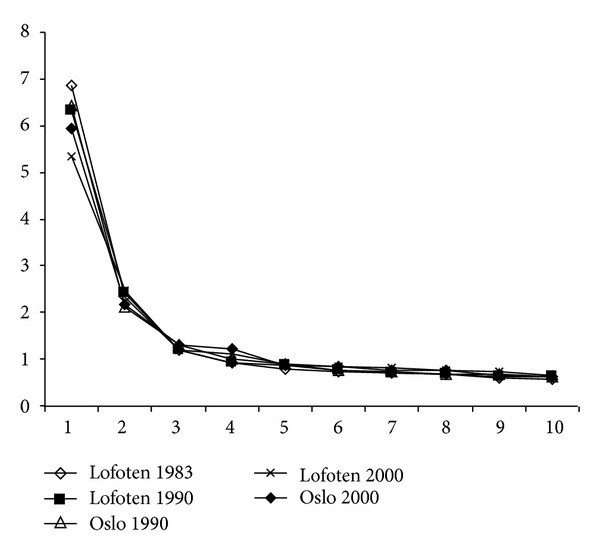
Scree plot for maximum likelihood factor analyses in five samples (eigenvalue as a function factor number in initial extraction).

Figure 1 shows a scree plot for the five analyses. In all analyses, there is a quite clear leveling off after three factors. A three-factor solution was therefore considered as adequate in all the samples.

Tables 2(a) and 2(b) show the factor scores for the first unrotated factor. It is not surprising that the Lofoten 1983 sample has most items loading high on this factor. When we chose items for the following surveys, one of the criteria was to have all the items that loaded highest on the first unrotated factor. The items that had the lowest loadings on the first unrotated factor in most/all of the samples are items using resources for mental health services and psychiatric patients. Seeing all five samples together, items with demand for every day coping and nondisturbing behavior seem to be the core of a main common dimension in the attitudes towards the mentally ill in these Norwegian surveys, stable over time and location; but also items indicating a wish for distance to psychiatric patients are fairly stable in the sense that they have high loadings on the first unrotated factor. Even if the rankings differ, the five highest loading items (A12, A19, A7, A2, and A4) are the same in Lofoten 1983, Lofoten 1990, Lofoten 2000, and Oslo 1990 samples.

The Oslo 2000 sample is to some degree different. Among the five items with highest loading on the first unrotated factor, three (A7, A12, and A19) are the same as for the other samples, and also the other two (A18 and A3) have loadings above 0.5 in the other four samples. Vice versa, the “missing” two (A2 and A4) from the other four samples also have high loadings in Oslo 2000. The additional two items with high loading on the first un-rotated factor in Oslo 2000 (A9 and A13) are related to location of psychiatric facilities in the neighborhood and a general tolerant attitude. But altogether the first unrotated factor in the Oslo 2000 sample is more similar than different compared to the four other samples.

Tables 3(a) and 3(b) show the oblique rotated factors from the five populations. In the tables, the items that load highest and at the same time do not load highly on another factor are marked in bold.

Factor 1 has items related to keeping distance to psychiatric patients. Six items (A11, A14 A15, A16, A17, and A18) are among the marked in all five analyses. The factor is named distance. Four of the six items are originally from Taylor and Dear.

Factor 2 has items that express a positive attitude, tolerance, and use of resources with regard to psychiatric patients. Three items (A8, A9, and A13) are among the marked in all five analyses. The factor is named positive. All three are from Taylor and Dear.

Factor 3 has items that mainly reflect demands that should be fulfilled by the patients before they could leave a psychiatric hospital and live in ordinary neighborhoods. Four items (A1, A2, A4, and A7) are among the marked in all five analyses. The factor is named demands. Only one item, A2, is from Taylor and Dear.

Table 4 shows the degree of fit between each specific sample and the factor structure obtained from analysis of pooled data from the remaining four samples. The chi-square values imply that the hypothesis of identical factor structures is clearly rejected in all cases. The goodness of fit measures, nevertheless, suggest that the deviations are not dramatic. Although no clear statistical criteria exist, values of CFI of above 0.90 and below 0.05 to 0.10 on the RMSEA are generally considered as acceptable (cf. Kline [26]).

### 5.2. Aim 3: Factor Structure of the 13 Common Attitude Items Used in Lofoten 1983, Lofoten 1990, Oslo 1990, Lofoten 2000, Oslo 2000, and Valdres 2010

**Figure 2:**
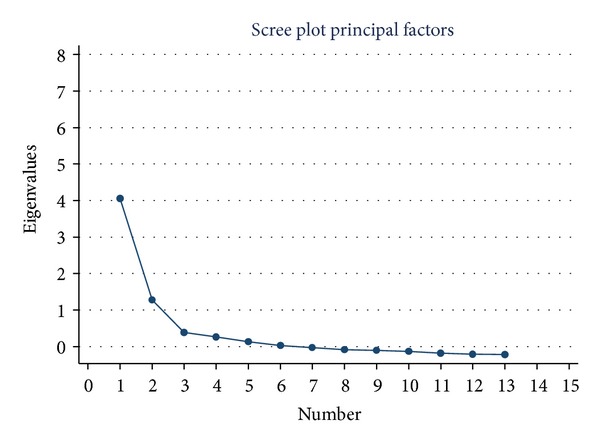
Valdres 2010.

Also, the scree plot from the Valdres sample (Figure 2) has a leveling off after three factors, resembling the five Lofoten-Oslo analyses, but the eigen-value for the third factor is below 1.

Using the 13 chosen items based on the series of factor analyses presented in Section 5.1, Tables 5(a), 5(b), and 5(c) compare the separate factor analyses of all six samples. The extraction method is principal factors, and a retained three-factor oblimin rotation is performed. Each subtable compares the six samples on one of the three factors/dimensions, that is, Distance, Demands, and Positive.

The Distance factor (Table 5(a)) has high loadings in all six samples on A11, A15, A16, and A17. A14 only loads high in the Lofoten 1983 sample. Even if A14 has its highest loading at the Distance factor for Lofoten 1990, Oslo 1990, Lofoten 2000, and Oslo 2000, they are all below 0.4. The loading for Valdres 2010 is very low, and here even loads highest on another factor (Positive, but below 0.4). A18 loads above 0.4 in Lofoten 1983, Lofoten 1990, Oslo 1990, and Lofoten 2000. Even if there are loadings about 0.3 in the Oslo-2000 and Valdres 2010 samples, they both have their highest loading on the Positive factor.

The Demands factor (Table 5(b)) has the high loadings on the same four items in all six samples, that is, A1, A2, A4, and A7.

The Positive factor (Table 5(c)) has the high loadings on the same predicted items in all six samples, that is, A8, A9, and A13. (In Lofoten 1990, A13 is just below 0.4.) Also, in the Oslo 2000 and Valdres 2010 samples, A18 and A19 have their highest loadings on the Positive factor.

The two items, A14 and A18, which alternate between Distance and Positive, are both related to location of psychiatric patients, that is, in hospitals or in ordinary neighborhoods.

### 5.3. Aim 4: Tests of Scale Reliabilities (Cronbach's Alpha)

 The indexes are based on the stable items revealed in the analyses of the five Lofoten/Oslo samples (see Result Section 5.1). They are constructed by adding the absolute value of each item included in the index. The Distance index consists of A11, A14, A15, A16, A17, and A18. The Demands index consists of A1, A2, A4, and A7. The Positive index consists of A8, A9, and A13. Cronbach's alpha level is acceptable for Distance, varying from 0.830 to 0.779 in the six samples; good for Demands, varying from 0.852 to 0.804; questionable for Positive, varying from 0.647 to 0.522 (see Tables 6(a) to 6(f) in Appendix B).

The Norwegian Institute of Public Health (NIPH) has picked nine items (to cover the three rotated dimensions) to be used in monitoring attitudes toward the mentally ill. Their items are A11, A15, and A17 (PH.Distance); A8, A9, and A13 (PH.Positive); A2, A4, and A7 (PH.Demand), all among the stable items from the present factor analyses.  Table 6(g) in Appendix B shows Cronbach's alpha for indexes using the items picked by NIPH. Cronbach's alpha level is questionable for PH.Distance, acceptable for PH.Demand, and questionable for PH.Positive.

## 6. Discussion

In the first part of the present study (Lofoten 1983, 1990, and 2000 and Oslo 1990 and 2000), we found three (oblimin) rotated factors that had an acceptable stability across time and place (urban-rural). In all five samples, 13 of the 19 items could consistently be associated with the same factor.

In the analyses of the 16 CAMI items in Lofoten 1983, a three-factor solution, or rather a two-factor solution, did fit the data set. With regard to the Taylor and Dear dimension, one factor covered authoritarianism and social restrictiveness, whereas benevolence and mental health ideology loaded on the second, especially if corrected for acquiescence effects [23]. In the factor analyses of the 13 items, using all six samples, the CAMI items did concentrate on two factors, that is, Distance and Positive, each with three original CAMI items. The third dimension, Demands, had only one CAMI item and mostly reflected the additional dimension we theoretically added in 1983, with items about the demands people made on psychiatric patients if they should be allowed to stay out of mental hospitals.

The fact that the CAMI items in essence yielded two main dimensions was in accordance with the roots of the Taylor and Dear questionnaire. Their point of departure was the Opinions of Mental Illness Questionnaire (OMI) [28, 29], which identified two distinct prejudicial attitudes toward mental illness, corresponding with authoritarianism and benevolence, dimensions that accounted for the greatest variance. This was also the case in a study by Brockington et al. [30]. In a path-analytic study, Corrigan et al. [31] found significant paths between these two factors and social distance (to mentally ill people). Angermeyer et al. [32, 33], using all 40 CAMI items, found four factors, fairly like the ones described by Taylor and Dear. A study from Israel [34] also found four factors, resembling the OMI structure of studies from USA [28], but no distinct social benevolence factor emerged in Israel. This was explained by Israel having no community psychiatry and deinstitutionalization policy at the time of the study. Also, Greek studies [18, 35] have used the OMI items. Compared to the original factor scores of Cohen and Struening, the Greek studies found different items that loaded on some of their five factors, but the patterns were not far removed from each other. In a British study by Wolf et al. [36, 37], done in connection with location of mental health facilities, the CAMI items were factor analyzed and yielded three factors only broadly similar to the factors from Taylor and Dear. However, Wolf et al.'s fear and exclusion, social control, and goodwill had some resemblance to Taylor and Dear's community mental health ideology, authoritarianism, and benevolence, but to a lesser extent social restrictiveness as well as also some likeness with Brockington et al.'s factors. Also, a recent American study among students mostly repeated the factor structures found earlier [38].

Papadopoulos et al. [39] did not factor analyze the CAMI items themselves, but in their study of UK-born Greek Cypriots and White English ethnicity population, they found strong reliability on each attitudinal scale by alpha-coefficient reliability test (authoritarianism = 0.64, benevolence = 0.73,  social restrictiveness = 0.78). A Japanese study [40] used different items but found one factor, fear of the mentally ill, resembling one of the factors of Brockington. A recent Swedish study [41] added behavioral items like the present study and revealed a related pattern, that is, four factors, intention to interact, fearful and avoidant, open-minded and prointegration, and community mental health ideology. Together, the referred studies, as well as the present study, have given strong indications of common attitude factors across different cultures.

One reason for instability regarding attitude factors across different studies could be related to change in the conceptualization of psychiatric patients, the definition of being mentally ill. Phelan et al. [42] compared surveys from 1950 and 1996 and found that conceptions of mental illness had broadened somewhat over this time period to include a greater proportion of nonpsychotic disorders. On the other hand, perceptions that mentally ill people were violent or frightening substantially increased rather than decreased. In the present studies, we have used an introduction that should include the same types of psychiatric patients across time. The one difference we found in Oslo 2000 could rather be explained by a marked increase in patients and institutions allocated to residential areas, more than that in Lofoten. Principally, the factor analyses in the Valdres sample, a new location surveyed 10 years later than the last Lofoten/Oslo survey, show a fairly similar pattern as the one found in the earlier analyses. The main dissimilarity is found in the Distance dimension which shared loadings with the Positive dimensions. A two-factor pattern may have been an alternative. The reason for the fairly small instability we found when we compared the 13 items in the six samples (Tables 5(a), 5(b), and 5(c)) may be that the restructuring of the Norwegian psychiatric service was finished, with less discussion about where psychiatric facilities and treatment of psychiatric patients should take place. One reason for the stability of factors may be the stability of stereotypes about mental illness, despite changes in treatment and social function of psychiatric patients [43]. Later studies have in addition to the dimensions revealed in the present studies had more explicit focus on stigmatizing attitudes towards the mentally ill [38, 44, 45].

## 7. Conclusion

Based on factor analyses of five Norwegian general population samples (Oslo, urban 1990 and 2000, and Lofoten, rural 1983, 1990, and 2000), newer research may choose to include the items that were stable in factor analyses and covered three central dimensions of attitudes towards the mentally ill. This was strengthened by analyses of a later sample (Valdres, rural 2010) that showed a similar pattern. Hence, the found dimensions, Distance, Demands, and Positive, seemed to be reasonably stable within a variety of Norwegian samples. On the other hand, the analyses were different enough to recommend researchers and politicians to be careful when comparing absolute levels of the suggested indexes across different locations and at different points in time. Words we have used may have changed contents and connotations. The relevance and meaning of a particular question could have been affected by structural changes of the psychiatric treatment system and not necessarily indicating changes in attitudes towards the mentally ill. However, the present studies contribute to the development of such comparable indexes. Even if all the populations that were covered were Norwegian, they included very different urban and rural areas and across a fairly long period of time.

## Figures and Tables

**Table 1:** Sample sizes. Lofoten, Oslo, and Valdres.

	1983	1990	2000	2010
Lofoten	470	947	864	
Oslo		948	467	
Valdres				772

**Table:** (a) Lofoten

Variable	1983	1990	2000
A1 Cook Before Leave	0.640	0.593	0.682
A2 Disturbed Hospitalized (T)	0.709	0.663	0.723
A3 Facilities Not In Dwelling (T)	0.632	0.600	0.628
A4 Find Their Way	0.709	0.767	0.755
A5 Difficult Understand	0.541	0.510	0.523
A6 Responsibility Care (T)	−0.024	−0.016	−0.091
A7 Keep Order Economy	0.734	0.758	0.738
A8 More Tax Money (T)	0.162	0.158	−0.077
A9 Accept Facilities (T)	−0.018	−0.157	−0.317
A10 Think About Appearance	0.651	0.592	0.516
A11 Keep Away	0.549	0.523	0.460
A12 Like Most People	0.761	0.730	0.747
A13 More Tolerant (T)	−0.063	−0.145	−0.306
A14 Too Much Money	0.500	0.407	0.328
A15 Not Responsibility (T)	0.631	0.575	0.520
A16 Foolish To Marry (T)	0.649	0.586	0.505
A17 Not Next Door (T)	0.633	0.570	0.591
A18 Resist Location (T)	0.585	0.517	0.607
A19 Attention Hospitalized	0.760	0.723	0.735

**Table:** (b) Oslo

Variable	1990	2000
A1 Cook Before Leave	0.568	0.507
A2 Disturbed Hospitalized (T)	0.604	0.559
A3 Facilities Not In Dwelling (T)	0.557	0.674
A4 Find Their Way	0.720	0.621
A5 Difficult Understand	0.430	0.438
A6 Responsibility Care (T)	−0.028	−0.126
A7 Keep Order Economy	0.682	0.631
A8 More Tax Money (T)	0.026	−0.182
A9 Accept Facilities (T)	−0.280	−0.514
A10 Think About Appearance	0.538	0.478
A11 Keep Away	0.379	0.455
A12 Like Most People	0.691	0.656
A13 More Tolerant (T)	−0.197	−0.473
A14 Too Much Money	0.391	0.386
A15 Not Responsibility (T)	0.455	0.487
A16 Foolish To Marry (T)	0.428	0.543
A17 Not Next Door (T)	0.511	0.594
A18 Resist Location (T)	0.552	0.725
A19 Attention Hospitalized	0.667	0.626

**Table:** (a)

Lofoten	1983			1990			2000		
Variable	Factor 1	Factor 2	Factor 3	Factor 1	Factor 2	Factor 3	Factor 1	Factor 2	Factor 3
A1 Cook Before Leave	−0.006	0.070	−**0.728**	−0.080	0.045	−**0.791**	−0.099	−0.006	−**0.864**
A2 Disturbed Hospitalized (T)	0.122	−0.101	**−0.717**	0.303	0.020	−**0.483**	0.207	−0.002	−**0.608**
A3 Facilities Not In Dwelling (T)	0.300	−0.049	−**0.471**	0.433	−0.024	−0.338	0.381	−0.124	−0.338
A4 Find Their Way	−0.169	0.028	−**0.939**	−0.050	0.024	−**0.917**	−0.102	0.033	−**0.932**
A5 Difficult Understand	0.111	0.075	−**0.531**	0.158	0.146	−**0.465**	0.177	0.047	−**0.448**
A6 Responsibility Care (T)	0.118	** 0.697**	0.049	0.052	** 0.684**	−0.043	0.028	**0.507**	−0.109
A7 Keep Order Economy	−0.072	0.061	−**0.855**	0.076	0.007	−**0.769**	0.086	0.064	**−0.729**
A8 More Tax Money (T)	0.045	** 0.684**	−0.059	0.030	** 0.584**	−0.161	0.056	** 0.657**	−0.055
A9 Accept Facilities (T)	−0.081	** 0.670**	−0.036	−0.071	** 0.715**	0.021	−0.041	** 0.722**	0.045
A10 Think About Appearance	0.130	0.105	−**0.565**	0.325	0.124	−0.356	0.394	0.153	−0.271
A11 Keep Away	**0.642**	0.060	−0.044	** 0.748**	0.048	0.022	** 0.665**	0.048	0.038
A12 Like Most People	0.254	−0.029	−**0.616**	0.440	−0.016	−0.410	0.429	0.013	−0.412
A13 More Tolerant (T)	−0.012	** 0.659**	0.000	0.021	** 0.738**	0.084	−0.026	** 0.616**	0.060
A14 Too Much Money	** 0.658**	0.064	−0.024	** 0.604**	0.039	0.010	** 0.478**	0.001	−0.035
A15 Not Responsibility (T)	** 0.683**	−0.020	−0.112	** 0.765**	0.000	0.012	** 0.666**	0.029	−0.004
A16 Foolish To Marry (T)	** 0.699**	0.044	−0.074	** 0.684**	0.082	−0.049	** 0.711**	0.159	0.048
A17 Not Next Door (T)	**0.830**	0.009	0.034	** 0.840**	−0.030	0.049	** 0.729**	−0.087	−0.003
A18 Resist Location (T)	** 0.786**	−0.019	0.005	** 0.795**	−0.033	0.048	** 0.708**	−0.190	−0.060
A19 Attention Hospitalized	0.278	0.003	**−0.560**	** 0.616**	0.002	−0.236	** 0.434**	−0.004	−0.402

**Table:** (b)

Oslo	1983			1990			2000		
Variable	Factor 1	Factor 2	Factor 3	Factor 1	Factor 2	Factor 3	Factor 1	Factor 2	Factor 3
A1 Cook Before Leave				−0.042	0.048	**−0.762**	−0.087	0.044	**−0.821**
A2 Disturbed Hospitalized (T)				0.266	0.127	**−0.499**	0.277	0.041	**−0.493**
A3 Facilities Not In Dwelling (T)				0.535	−0.061	−0.249	0.323	−0.384	−0.377
A4 Find Their Way				−0.049	−0.019	**−0.926**	−0.052	−0.022	**−0.866**
A5 Difficult Understand				0.304	0.106	−0.332	** 0.422**	0.014	−0.134
A6 Responsibility Care (T)				0.239	** 0.586**	0.017	0.063	0.319	−0.083
A7 Keep Order Economy				0.037	0.048	**−0.767**	0.194	0.075	**−0.632**
A8 More Tax Money (T)				0.024	**0.632**	−0.130	0.008	** 0.470**	−0.115
A9 Accept Facilities (T)				−0.144	** 0.786**	−0.025	0.000	** 0.794**	0.147
A10 Think About Appearance				0.264	0.171	−0.399	** 0.464**	0.063	−0.197
A11 Keep Away				** 0.623**	0.205	−0.021	** 0.671**	0.048	0.086
A12 Like Most People				0.428	−0.004	−0.427	** 0.520**	0.003	−0.301
A13 More Tolerant (T)				0.009	** 0.723**	0.031	−0.055	** 0.512**	0.050
A14 Too Much Money				** 0.585**	0.003	−0.070	** 0.495**	−0.049	0.046
A15 Not Responsibility (T)				** 0.702**	0.086	−0.002	** 0.658**	0.086	0.033
A16 Foolish To Marry (T)				** 0.690**	0.083	0.008	** 0.663**	0.035	−0.010
A17 Not Next Door (T)				** 0.869**	−0.033	0.078	** 0.758**	−0.111	0.110
A18 Resist Location (T)				** 0.825**	−0.125	−0.015	** 0.598**	−0.371	−0.101
A19 Attention Hospitalized				** 0.477**	0.016	−0.376	** 0.585**	0.029	−0.210

**Table 4:** Goodness of fit for each sample of factor model derived from pooled analyses of the four remaining samples.

	Chi-square (171 d.f.)	RMSEA	CFI
Lofoten 1983	413.266	0.058	0.970
Lofoten 1990	765.187	0.063	0.958
Oslo 1990	1032.018	0.071	0.916
Lofoten 2000	1023.348	0.076	0.937
Oslo 2000	981.203	0.074	0.940

**Table:** (a) Distance factor

Location						
Variable	Lofoten T0	Lofoten T1	Oslo T1	Lofoten T2	Oslo T2	Valdres
A1	0.034	−0.124	−0.086	−0.077	−0.055	−0.105
A2	0.231	0.303	0.271	0.261	0.195	0.244
A4	−0.072	0.010	−0.029	−0.054	−0.077	0.028
A7	0.040	0.114	0.090	0.129	0.170	0.027
A8	0.084	0.087	0.039	0.060	0.032	0.135
A9	−0.077	−0.110	−0.206	−0.052	0.071	−0.036
A11	0.619	0.639	0.431	0.539	0.513	0.403
A13	−0.010	−0.093	−0.087	−0.056	−0.159	−0.078
A14	0.572	0.383	0.294	0.255	0.243	0.025
A15	0.626	0.664	0.541	0.587	0.519	0.438
A16	0.694	0.662	0.612	0.685	0.659	0.616
A17	0.749	0.741	0.658	0.590	0.558	0.738
A18	0.695	0.580	0.473	0.457	0.298	0.339

**Table:** (b) Demands factor

Location						
Variable	Lofoten T0	Lofoten T1	Oslo T1	Lofoten T2	Oslo T2	Valdres
A1	0.696	0.766	0.732	0.805	0.775	0.763
A2	0.516	0.412	0.443	0.552	0.498	0.473
A4	0.855	0.827	0.780	0.845	0.808	0.813
A7	0.730	0.719	0.682	0.699	0.674	0.735
A8	0.049	0.091	0.076	0.091	0.159	0.082
A9	0.012	−0.043	−0.031	−0.036	−0.050	−0.014
A11	−0.028	−0.046	−0.021	0.003	−0.038	0.065
A13	−0.064	−0.051	−0.019	−0.063	−0.011	−0.023
A14	−0.014	0.115	0.130	0.051	−0.016	0.248
A15	0.105	0.032	0.029	0.031	0.043	0.060
A16	0.025	0.066	−0.017	−0.009	0.084	0.036
A17	−0.018	−0.025	−0.019	0.054	−0.032	−0.034
A18	−0.008	0.020	0.096	0.098	0.096	0.080

**Table:** (c) Positive factor

Location						
Variable	Lofoten T0	Lofoten T1	Oslo T1	Lofoten T2	Oslo T2	Valdres
A1	0.001	−0.022	−0.034	−0.033	0.010	−0.032
A2	−0.050	0.060	0.192	0.038	0.009	0.141
A4	0.007	0.008	−0.040	−0.011	−0.055	0.007
A7	0.022	0.017	0.075	0.055	0.038	−0.026
A8	0.562	0.542	0.554	0.561	0.443	0.516
A9	0.553	0.557	0.482	0.584	0.694	0.694
A11	0.004	0.069	0.088	−0.033	0.007	−0.221
A13	0.486	0.376	0.416	0.409	0.407	0.658
A14	−0.030	−0.123	−0.245	−0.184	−0.236	−0.357
A15	0.003	−0.013	0.056	−0.021	0.002	−0.060
A16	0.089	0.125	0.054	0.126	0.042	0.027
A17	0.011	−0.070	−0.111	−0.148	−0.271	−0.058
A18	−0.096	−0.183	−0.257	−0.341	−0.522	−0.416

**Table:** (a) Lofoten T0

Variable	Average interitem covariance	Number of items in the scale	Scale reliability coefficient
Distance	0.750	6	0.830
Positive	0.256	3	0.576
Demands	0.961	4	0.824

**Table:** (b) Lofoten T1

Variable	Average interitem covariance	Number of items in the scale	Scale reliability coefficient
Distance	0.651	6	0.801
Positive	0.226	3	0.522
Demands	0.919	4	0.820

**Table:** (c) Oslo T1

Variable	Average interitem covariance	Number of items in the scale	Scale reliability coefficient
Distance	0.371	6	0.718
Positive	0.254	3	0.538
Demands	0.839	4	0.804

**Table:** (d) Lofoten T2

Variable	Average interitem covariance	Number of items in the scale	Scale reliability coefficient
Distance	0.448	6	0.748
Positive	0.309	3	0.563
Demands	1.024	4	0.852

**Table:** (e) Oslo T2

Variable	Average interitem covariance	Number of items in the scale	Scale reliability coefficient
Distance	0.373	6	0.739
Positive	0.255	3	0.543
Demands	0.807	4	0.819

**Table:** (f) Valdres

Variable	Average interitem covariance	Number of items in the scale	Scale reliability coefficient
Distance	0.298	6	0.779
Positive	0.242	3	0.647
Demand	0.489	4	0.807

**Table:** (g) Cronbach's alpha on PH.Distance, PH.Positive, and PH.Demand. Test scale = mean (unstandardized items)

Variable	Average interitem covariance	Number of items in the scale	Scale reliability coefficient
PH.Distance	0.289	3	0.653
PH.Positive	0.242	3	0.647
PH.Demand	0.473	3	0.747

## Appendices

### A. The 19 Statements Common for the First Five Surveys

The 19 statements common for the first five surveys:

(T) items taken from Taylor and Dear (1981);

(V) items chosen for the Valdres survey;

(PH) items chosen by Norwegian Institute of Public Health.

The self-completing questionnaire has the following introduction. People have different experience with long-term psychiatric patients. We are therefore interested to hear your opinion about different aspects concerning such patients. Next, there are a set of statements we want you to take an attitude towards. With a psychiatric patient, we understand a person who has been treated for mental illness, and has been hospitalized in a psychiatric hospital (in Valdres, this is phrased 24 hour institution).

1. Psychiatric patients must be able to cook and keep their clothes and house in good order, before they can leave the hospital. (V) A1 COOK BEFORE LEAVE.
2. As soon as a person shows signs of mental disturbance, he should be hospitalized. (T) (V) (PH) A2 DISTURBED HOSPITALIZED.
3. Mental health facilities should be kept out of residential neighborhoods. (T) A3 FACILITIES NOT IN DWELLING.
4. Psychiatric patients must be able to find their way to shops, public offices, and pick their way with bus, boats, and so forth, before they can leave the hospital. (V) (PH) A4 FIND THEIR WAY.
5. It is difficult to understand psychiatric patients. One never knows what they think, plan, or react to. A5 DIFFICULT UNDERSTAND.
6. We have a responsibility to provide the best possible care for psychiatric patients. (T) A6 RESPONSIBILITY CARE.
7. Psychiatric patients must be able to keep order in their personal economy before they can leave the hospital. (V) (PH) A7 KEEP ORDER ECONOMY.
8. More tax money should be spent on the care and treatment for psychiatric patients. (T) (V) (PH) A8 MORE TAX MONEY.
9. Residents should accept the location of mental health facilities in their neighborhood to serve the needs of the local community. (T) (V) (PH) A9 ACCEPT FACILITIES.
10. Psychiatric patients should think about their appearance and how they tidy and dress themselves. A10 THINK ABOUT APPEARANCE.
11. One should keep away from everyone with psychiatric problems. (V) (PH) A11 KEEP AWAY.
12. Psychiatric patients must be like most people before they can leave the hospital. A12 LIKE MOST PEOPLE.
13. We need to adopt a far more tolerant attitude toward psychiatric patients in our society. (T) (V) (PH) A13 MORE TOLERANT.
14. To day, one uses too much money to get people from the psychiatric hospitals back to their home communities. (V) A14 TO MUCH MONEY.
15. Psychiatric patients should not be given any responsibility. (T) (V) (PH) A15 NOT RESPONSIBILITY.
16. A woman would be foolish to marry a man who has suffered from mental illness, even though he seems fully recovered. (T) (V) A16 FOOLISH TO MARRY.
17. I would not want to live next door to someone who has been mentally ill. (T) (V) (PH) A17 NOT NEXT DOOR.
18. Local residents have good reason to resist the location of mental health services in their neighborhood. (T) (V) A18 RESIST LOCATION.
19. Psychiatric patients that attract attention should be hospitalized as soon as possible. A19 ATTENTION HOSPITALIZED.

### B. Cronbach's Alpha on Attitude Dimensions

See Tables 6(a), 6(b), 6(c), 6(d), 6(e), 6(f), and 6(g).
